# Reduced Hospitalizations and Amputations in Patients with Diabetic Foot Ulcers Treated with Cyclical Pressurized Topical Wound Oxygen Therapy: Real-World Outcomes

**DOI:** 10.1089/wound.2021.0118

**Published:** 2022-09-15

**Authors:** Jessica Izhakoff Yellin, Julia A. Gaebler, Frank F. Zhou, Timothy Niecko, Olivia Novins, Amelia Ockert, Darcy Krzynowek, Matthew G. Garoufalis, Aliza M. Lee, Robert G. Frykberg

**Affiliations:** ^1^ Health Advances LLC, Newton, Massachusetts, USA.; ^2^ Niecko Health Economics LLC, Tierra Verde, Florida, USA.; ^3^ Department of Podiatry, Jesse Brown VA Medical Center, Chicago, Illinois, USA.; ^4^ Department of Podiatry, Salem Veterans Affairs Medical Center, Salem, Virginia, USA.; ^5^ Department of Podiatry, Diabetic Foot Consultants, Midwestern University, Glendale, Arizona, USA.

**Keywords:** diabetic foot ulcers, topical oxygen therapy, amputations, hospitalizations

## Abstract

**Background:**

This study sought to examine the real-world impact of multimodality cyclical-pressure topical wound oxygen therapy (TWO2) on hospitalizations and amputations in patients with diabetic foot ulcer (DFU) compared with patients without TWO2.

**Methods:**

We conducted a retrospective review of deidentified patient medical records at 2 U.S. Veterans Affairs hospitals between January 2012 and January 2020. DFU patients were assigned to TWO2 or NO TWO2 cohorts based on their treatment records. Patients received appropriate standard of care and may have received other advanced wound treatments, including skin substitutes, negative pressure wound therapy, and growth factors. Primary study outcomes were patients requiring hospitalization and/or amputation within 360 days of initial wound documentation.

**Findings:**

Among unmatched cohorts of 202 patients with DFU (91 TWO2, 111 NO TWO2), 6.6% and 12.1% of TWO2 patients had hospitalizations and amputations, respectively, compared with 54.1% and 41.4% of NO TWO2 patients within 360 days (*p* < 0.0001, *p* < 0.0001), representing 88% and 71% reductions. Among propensity score-matched cohorts of 140 DFU patients (70 TWO2, 70 NO TWO2), compared with NO TWO2, 82% fewer TWO2 patients were hospitalized (7.1% vs. 40.0%, *p* < 0.0001) and 73% fewer TWO2 patients had amputations (8.6% vs. 31.4%, *p* = 0.0007). Logistic regression among matched cohorts demonstrated nearly ninefold and fivefold higher risk of hospitalization and amputation, respectively, for NO TWO2 versus TWO2.

**Interpretation:**

This retrospective cohort study demonstrates that treating patients with DFU with TWO2 is associated with significant reductions in hospitalizations and amputations in the real-world setting.

**Figure:**
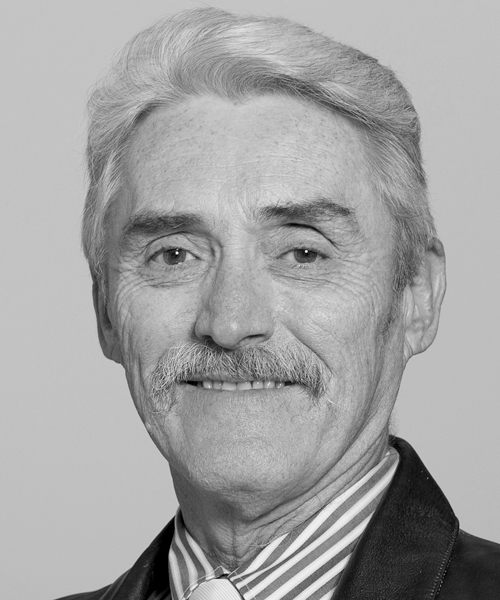
Robert G. Frykberg, DPM, MPH

## Introduction

Diabetic foot ulcers (DFUs) are concerning, particularly as nonhealing and recurrent DFUs can lead to hospitalization, amputation, and death.^1,2^ Estimates of the 5-year mortality rate after lower extremity amputation range from 46% to 57%, depending on the type of amputation.^3^ As a result, DFUs continue to be a serious and costly condition.^4,5^ A 2014 study estimated that DFUs cost U.S. public and private payers $9–13 billion per year in addition to costs associated with diabetes.^6^

Standard of care (SOC) treatment for DFUs is widely accepted to include debridement, effective offloading, treatment of infection, and vascular intervention as required.^7,8^ However, DFU is a complex condition and despite optimal SOC, many wounds remain difficult to heal or frequently recur.^2^ While a number of adjunctive therapies are available for use, the quality of evidence about their respective efficacy rates is regarded to be low, due primarily to small and poorly designed studies, as well as difficulty extrapolating drawn conclusions to the general, real-world DFU population.^9,10^

Oxygen therapy has been widely studied in the treatment of DFU, as oxygen is critical to the process of healing a wound.^11^ Nonetheless, efficacy results from studies of hyperbaric oxygen therapy and topical oxygen therapy (TOT) vary, and conclusions regarding effectiveness are inconsistent.^12–14^ A recent double-blind, sham-controlled trial, however, showed significantly improved healing of chronic DFUs with multimodal cyclical pressurized topical wound oxygen therapy (TWO2) at both 12 weeks and 12 months.^15^

There are several different types of TOTs, each with unique properties in terms of dressings and/or mode of delivery of oxygen topically to the wound bed. These can be categorized as (1) normobaric flow of continuous diffusion of oxygen under proprietary dressing devices, (2) low constant pressure devices in a contained chamber, and (3) the device used in this study, incorporating cyclically pressurized (10–50 mb) and humidified oxygen delivery within a contained chamber or boot.^11^

Unfortunately, there is a paucity of studies to support the use of TOT within real-world, representative populations. This study, therefore, sought to evaluate the real-world impact of home-based cyclical pressurized topical wound oxygen therapy on DFUs by analyzing subsequent hospitalizations and amputations in a large, real-world, representative patient population.

## Methods

### Study population

We used deidentified data collected retrospectively from patient medical records at two U.S. Veterans Affairs (VA) hospitals. The period of review was January 2012 through January 2020. Patients were identified through a primary diagnosis of diabetic, ischemic, venous, pressure, and multimorbid wounds (total *N* = 246). Only patients with a primary diagnosis of DFU (either alone or multimorbid with other wounds) were included in the analysis (*N* = 202). The protocol was approved by the Institutional Review Board at each facility.

Patient medical records were reviewed for demographic information such as age, sex, and ethnicity, wound characteristics, including Wagner classification, wound duration, area, and additional clinical characteristics, including prior amputation, type 1 or 2 diabetes, neuropathy, cardiovascular disease (CVD), peripheral arterial disease (PAD), venous disease, pain level, and stage of kidney disease.

### Therapeutic interventions

We evaluated the impact of treating chronic DFU with home-based multimodality cyclical pressure topical wound oxygen therapy (TWO2), (AOTI Ltd., Galway, Ireland), on hospitalizations and amputations. As is common in clinical practice, study participants may have also received additional adjunctive therapies, including negative pressure wound therapy (NPWT), skin substitutes (SS), and/or growth factors (GF). This study focused on the impact of TWO2 in the real-world setting to corroborate the recent positive findings from the aforementioned strictly controlled randomized trial of TWO2 versus sham-treated controls.^15^

To evaluate the effectiveness of TWO2, we developed two comparison groups. Comparison #1 (C1) compared patients who had ever received TWO2 (TWO2) with those who had never received TWO2 (NO TWO2). Patients in each cohort may have received treatment with additional adjunctive therapies, so TWO2 was considered additive to adjunctive treatment in C1. Comparison #2 (C2) compared patients who had received only TWO2 and no other adjunctive wound care therapies (TWO2 ONLY) with those receiving SS, NPWT, and/or GF, but *not* TWO2 (OTHER TX ONLY), thus evaluating the impact of TWO2 in lieu of other adjunctive treatments. Patients in each cohort received appropriate SOC irrespective of any adjunctive therapies, including TWO2.

### Study outcomes

The primary study outcomes were defined as patients with one or more wound-related hospitalization or amputation within a 1-year analysis period. Medical records were reviewed for presence of wound-related hospitalization or amputation at 90, 180, and 360 days after first documentation of the wound. The presence of a first hospitalization or amputation at any time point before or at 360 days classified a patient as having had a hospitalization or amputation within 360 days. Because the study outcomes were patients with wound-related hospitalizations and amputations, no patient was counted for more than one hospitalization or amputation if multiple such episodes occurred.

### Statistical analysis

Missing data for demographics and clinical characteristics was imputed by the single imputation hot-deck method. This method uses observed values from the sample to impute (fill-in) missing values. In instances of missing outcomes data, we applied the last observation carried forward (LOCF) method, which is a common statistical approach to account for missing follow-up observations.^16^ We assumed that any patient without follow-up data suggesting a hospitalization or amputation during the 360-day period was categorized as not hospitalized or not amputated.

Baseline demographics and clinical characteristics were assessed by chi-square test for categorical and *t*-tests for continuous variables. In cases where cell size was small (<5) for categorical assessments chi-square may not be a valid test, thus Fisher's exact test was used.

When using an observational study design, such as a retrospective cohort study, subjects are not randomized to a treatment or control group. Confounding can occur when some of the covariates are related to both the treatment and the outcome. Consequently, there can be systematic differences between the treated subjects and the control subjects.

In the presence of confounding, statistical approaches are required to remove the effects of confounding when estimating the effect of the treatment. Propensity score matching minimizes the effects of confounding by achieving more balanced covariates in the absence of a randomized study design.^17,18^ We, therefore, applied propensity score matching by means of a greedy algorithm to match TWO2 patients to NO TWO2 patients in a 1:1 ratio. Cohorts were matched on age, sex, ethnicity, wound severity, prior amputation, use of offloading, and use of NPWT, SS, or GF.

In addition, the study dichotomous outcomes of wound-related hospitalization versus no hospitalization, and amputation versus no amputation were assessed by logistic regression within the matched cohorts for C1 (NO TWO2 vs. TWO2). The logistic model calculated odds ratios (ORs), 95% confidence intervals (CIs), and *p*-values for the study treatment arms (NO TWO2 vs. TWO2). Statistical tests were two-sided, and significance level was set at *p* < 0.05. Analyses were performed using SAS software version 9.4.

### Role of the funding source

The funders of the study had no role in study design, data analysis, data interpretation, or writing of the report, but did help coordinate data collection. All authors had full access to all the data in the study and had final responsibility for the decision to submit for publication.

## Results

### Study population

A total of 202 patients with DFU were identified. For C1, 91 DFU patients qualified as ever receiving TWO2 (TWO2), and 111 patients qualified as never receiving TWO2 (NO TWO2). During the 1-year analysis time frame, patients in each cohort may have been treated with other advanced wound care treatments, including SS, NPWT, and GF. For C2, 58 DFU patients received only TWO2 and no other adjunctive wound care therapies (TWO2 ONLY), and 34 patients received only SS, NPWT, and/or GF, but not TWO2 (OTHER TX ONLY).

Table 1 presents demographic and clinical characteristics of unmatched and matched cohorts. For C1, unmatched cohorts of TWO2 versus NO TWO2 were similar on most characteristics, including the use of adjunctive therapies, although statistically significant differences were present for wound severity (*p* < 0.0001), prior amputation (*p* = 0.011), pain level (*p* = 0.011), and stage of kidney disease (*p* < 0.0001). For C2, unmatched cohorts of TWO2 ONLY versus OTHER TX ONLY were also similar on most characteristics, differing on use of adjunctive therapies, as well as wound severity (*p* = 0.0005), pain level (*p* = 0.026), stage of kidney disease (*p* = 0.0008), and HbA1c (*p* = 0.038).

**Table 1.** Baseline demographics and clinical characteristics of unmatched and matched cohorts

	Unmatched Cohorts						Propensity Score Matched Cohorts		
	Comparison #1 (C1)			Comparison #2 (C2)			Comparison #1 (C1)		
	TWO2* N* = 91	NO TWO2* N* = 111	*p*	TWO2 ONLY* N* = 58	OTHER TX ONLY* N* = 34	*p*	TWO2 N = 70	NO TWO2* N* = 70	*p*
Patient demographics									
Age (years), mean (SD)	66.9 (9.9)	67.0 (8.7)	0.93	67.7 (11.2)	65.9 (6.5)	0.33	67.5 (9.7)	67.7 (8.4)	0.89
Sex, male, *n* (%)	89 (97.8)	107 (96.4)	0.69	58 (100.0)	34 (100.0)	NA	68 (97.1)	69 (98.6)	1.0
Ethnicity, *n* (%)			0.092			0.084			0.61
White	51 (56.0)	49 (44.1)		33 (56.9)	13 (38.2)		37 (52.9)	34 (48.6)	
Non-White	40 (44.0)	62 (55.9)		25 (43.1)	21 (61.8)		33 (47.1)	36 (51.4)	
Wound characteristics									
Wound classification, *n* (%)			<0.0001			0.0005			1.0
Wagner 1	39 (42.9)	38 (34.2)		29 (50.0)	8 (23.5)		30 (42.9)	30 (42.9)	
Wagner 2	45 (49.5)	39 (35.1)		27 (46.6)	15 (44.1)		33 (47.1)	33 (47.1)	
Wagner 3	5 (5.5)	25 (22.5)		1 (1.7)	8 (23.5)		5 (7.1)	4 (5.7)	
Wagner 4	2 (2.2)	9 (8.1)		1 (1.7)	3 (8.8)		2 (2.9)	3 (4.3)	
Presenting area (cm^2^), mean (SD)	3.8 (6.8)	3.9 (7.1)	0.95	3.2 (5.2)	7.2 (11.1)	0.056	3.3 (6.7)	3.6 (7.8)	0.80
Wound duration (days), mean (SD)	175.3 (241.7)	166.9 (240.8)	0.81	195.6 (271.0)	267.4 (316.1)	0.27	194.8 (269.8)	188.6 (261.4)	0.89
Use of other treatments									
Used offloading devices, *n* (%)	81 (89.0)	97 (87.4)	0.72	51 (87.9)	30 (88.2)	1.0	61 (87.1)	61 (87.1)	1.0
Used NPWT, *n* (%)	2 (2.2)	9 (8.1)	0.12	0 (0.0)	9 (26.5)	<0.0001	2 (2.9)	2 (2.9)	1.0
Used SS, *n* (%)	31 (34.1)	25 (22.5)	0.068	0 (0.0)	25 (73.5)	<0.0001	19 (27.1)	19 (27.1)	1.0
Used GF, *n* (%)	24 (26.4)	22 (19.8)	0.27	0 (0.0)	22 (64.7)	<0.0001	16 (22.9)	14 (20.0)	0.68
Other clinical characteristics									
Prior amputation, *n* (%)	41 (45.1)	31 (27.9)	0.011	21 (36.2)	11 (32.4)	0.71	20 (28.6)	20 (28.6)	1.0
Type 1 diabetes, *n* (%)	5 (5.5)	4 (3.6)	0.73	5 (8.6)	0 (0.0)	0.15	2 (2.9)	2 (2.9)	1.0
Type 2 diabetes, *n* (%)	86 (94.5)	107 (96.4)		53 (91.4)	34 (100.0)		68 (97.1)	68 (97.1)	
Neuropathy, *n* (%)	88 (96.7)	107 (96.4)	1.0	56 (96.6)	33 (97.1)	1.0	67 (95.7)	67 (95.7)	1.0
Cardiovascular disease, *n* (%)	69 (75.8)	79 (71.2)	0.46	45 (77.6)	27 (79.4)	0.84	51 (72.9)	51 (72.9)	1.0
Peripheral artery disease, *n* (%)	72 (79.1)	90 (81.1)	0.73	50 (86.2)	26 (76.5)	0.23	55 (78.6)	54 (77.1)	0.84
Peripheral edema, *n* (%)	22 (24.2)	31 (27.9)	0.55	14 (24.1)	9 (26.5)	0.80	15 (21.4)	18 (25.7)	0.55
Venous disease, *n* (%)	36 (39.6)	36 (32.4)	0.29	27 (46.6)	12 (35.3)	0.29	28 (40.0)	27 (38.6)	0.86
Pain level, mean (SD)	1.4 (2.3)	2.4 (3.3)	0.011	1.5 (2.1)	3.0 (3.5)	0.026	1.5 (2.4)	2.4 (3.3)	0.093
Kidney disease stage, *n* (%)			<0.0001			0.0008			<0.0001
Normal	1 (1.1)	19 (17.1)		0 (0.0)	4 (11.8)		1 (1.4)	15 (21.4)	
Stage I/II	23 (25.3)	24 (21.6)		15 (25.9)	6 (17.7)		18 (25.7)	11 (15.7)	
Stage III	45 (49.5)	31 (27.9)		32 (55.2)	8 (23.5)		36 (51.4)	21 (30.0)	
Stage IV/V	12 (13.2)	16 (14.4)		5 (8.6)	7 (20.6)		9 (12.9)	11 (15.7)	
Dialysis	10 (11.0)	21 (18.9)		6 (10.3)	9 (26.5)		6 (8.6)	12 (17.1)	
Diabetes duration (years), mean (SD)	17.4 (9.9)	18.0 (9.0)	0.66	17.5 (11.5)	18.9 (7.1)	0.48	17.4 (9.8)	17.4 (8.8)	0.99
HbA1c, mean (SD)	8.0 (1.7)	8.6 (2.2)	0.053	7.9 (1.8)	8.8 (2.1)	0.038	7.8 (1.7)	8.7 (2.5)	0.0093

Comparison #1 (C1) compares patients who ever received TWO2 (TWO2) to those who never received TWO2 (NO TWO2). Patients in both cohorts may have also received other adjunctive therapy. Comparison #2 (C2) compares patients who only received TWO2 and no other adjunctive therapy (TWO2 ONLY) to those who received NPWT, SS, and/or GF, but not TWO2 (OTHER TX ONLY). Propensity score matching was performed on the following 9 factors; age, sex, ethnicity, wound severity, prior amputation, use of offloading, use of NPWT, use of SS, use of GF. All patients received appropriate SOC.

GF, growth factors; NPWT, negative pressure wound therapy; SD, standard deviation; SOC, standard of care; SS, skin substitutes.

Similarly, C1 is tabulated after application of propensity score matching for age, sex, ethnicity, wound severity, prior amputation, use of offloading, and use of NPWT, SS, or GF. After propensity score matching, the TWO2 (*n* = 70) and NO TWO2 (*n* = 70) cohorts were well matched on all demographics and clinical characteristics, except for kidney disease (*p* < 0.0001) and HbA1c (*p* = 0.0093).

Most of the matched patients were male (97.1% TWO2, 98.6% NO TWO2), and approximately half of patients were white (52.9% TWO2, 48.6% NO TWO2). Many patients also had prior amputation (28.6% TWO2, 28.6% NO TWO2). A large proportion of patients in each cohort also had CVD (72.9% TWO2, 72.9% NO TWO2), PAD (78.6% TWO2, 77.1% NO TWO2), and venous disease (40.0% TWO2, 38.6% NO TWO2). On average, patients in each cohort had a mean diabetes duration of 17.4 years. Average wound duration was similar in the matched cohorts at 194.8 days for TWO2 and 188.6 days for NO TWO2 patients.

During the 12-month observation period, we only had one mortality in the NO TWO2 cohort. There were no instances of mortality in the TWO group.

### Outcomes for unmatched cohorts

**Figure 1.**
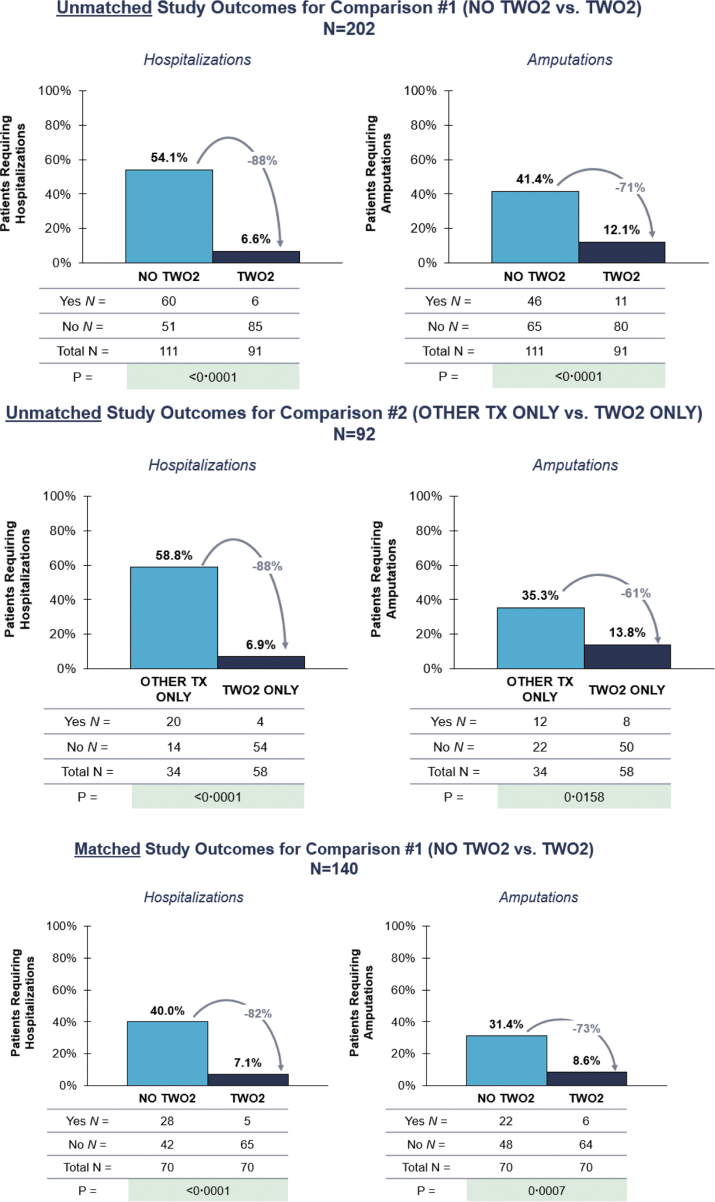
Matched and unmatched study outcomes. Comparison #1 (C1) compares patients who ever received TWO2 (TWO2) to those who never received TWO2 (NO TWO2). Patients in both cohorts may have also received other adjunctive therapy. Comparison #2 (C2) compares patients who only received TWO2 and no other adjunctive therapy (TWO2 ONLY) to those who received NPWT, SS, and/or GF, but not TWO2 (OTHER TX ONLY). Propensity score matching was performed on the following 9 factors; age, sex, ethnicity, wound severity, prior amputation, use of offloading, use of NPWT, use of SS, and use of GF. All patients received appropriate SOC. GF, growth factor; NPWT, negative pressure wound therapy; SOC, standard of care; SS, skin substitutes.

Table 2 presents outcomes for all cohorts. Within unmatched cohorts for C1, compared with NO TWO2, the proportion of TWO2 patients requiring hospitalization and amputation was 88% lower (6.6% vs. 54.1%, *p* < 0.0001) and 71% lower (12.1% vs. 41.4%, *p* < 0.0001), respectively, within 360 days (Fig. 1).

**Table 2.** Patients with hospitalization and amputation across cohorts

	Unmatched Cohorts						Propensity Score*–*Matched Cohorts		
	Comparison #1 (C1)			Comparison #2 (C2)			Comparison #1 (C1)		
	TWO2* N* = 91	NO TWO2* N* = 111	*p*	TWO2 ONLY* N* = 58	OTHER TX ONLY* N* = 34	*p*	TWO2* N* = 70	NO TWO2* N* = 70	*p*
Patients with hospitalization, *n* (%)	6 (6.6)	60 (54.1)	<0.0001	4 (6.9)	20 (58.8)	<0.0001	5 (7.1)	28 (40.0)	<0.0001
Patients with amputation, *n* (%)	11 (12.1)	46 (41.4)	<0.0001	8 (13.8)	12 (35.3)	0.016	6 (8.6)	22 (31.4)	0.0007

Comparison #1 (C1) compares patients who ever received TWO2 (TWO2) to those who never received TWO2 (NO TWO2). Patients in both cohorts may have also received other adjunctive therapy. Comparison #2 (C2) compares patients who only received TWO2 and no other adjunctive therapy (TWO2 ONLY) to those who received NPWT, SS, and/or GF, but not TWO2 (OTHER TX ONLY). Propensity score matching was performed on the following 9 factors; age, sex, ethnicity, wound severity, prior amputation, use of offloading, use of NPWT, use of SS, use of GF. All patients received appropriate SOC.

Within unmatched cohorts for C2, in contrast with OTHER TX ONLY, the proportion of TWO2 ONLY patients with a wound-related hospitalization was 88% lower (6.9% vs. 58.8%, *p* < 0.0001) and the proportion with amputation was 61% lower (13.8% vs. 35.3%, *p* = 0.016) within 360 days (Fig. 1).

### Outcomes for matched cohorts

Within matched cohorts, TWO2 patients still experienced reduced hospitalizations and amputations versus NO TWO2 within 360 days (Table 2). Compared with NO TWO2, 82% fewer TWO2 patients were hospitalized (7.1% vs. 40.0%, *p* < 0.0001) and 73% fewer TWO2 patients had an amputation (8.6% vs. 31.4%, *p* = 0.0007) (Fig. 1).

### Regression models in matched cohorts

Logistic regression models were conducted on the dichotomous study outcomes (hospitalization vs. no hospitalization, and amputation vs. no amputation) within matched cohorts of NO TWO2 and TWO2 patients. The models demonstrated a nearly ninefold greater risk of wound-related hospitalization (OR: 8.667; 95% CI: 3.101, 24.219; *p* < 0.0001) and nearly fivefold greater risk of amputation (OR: 4.887; 95% CI: 1.840–12.985; *p* = 0.0015) for NO TWO2 patients compared with TWO2 within 360 days (Table 3).

**Table 3.** Logistic regression models for matched cohorts

	Comparison #1 (C1)		
	OR	95% CI	*p*
Hospitalization (NO TWO2 vs. TWO2)	8.667	(3.101, 24.219)	<0.0001
Amputation (NO TWO2 vs. TWO2)	4.887	(1.840, 12.985)	0.0015

Study outcomes were assessed through logistic regression models for propensity score–matched cohorts in Comparison #1 (C1). C1 compares patients who ever received TWO2 (TWO2) to those who never received TWO2 (NO TWO2). Patients in both cohorts may have also received other adjunctive therapy. Propensity score matching was performed on the following 9 factors; age, sex, ethnicity, wound severity, prior amputation, use of offloading, use of NPWT, use of SS, use of GF.

CI, confidence interval; OR, odds ratio.

## Discussion

This study demonstrates the real-world effectiveness of cyclical pressurized topical wound oxygen therapy in reducing wound-related hospitalizations and amputations for patients with DFU compared with patients who did not receive this intervention. Both amputations and hospitalizations have been shown to contribute substantially to the overall cost burden of an ulcerated patient.^4–6^

Studies have also demonstrated that the annual cost of care for DFU is higher in patients with amputation due to increased health care utilization, such as increased provider visits, rehabilitation care, and other medical expenses.^19^ In 2010, Franklin *et al.* estimated such costs within the VA to be $60,647 per patient.^20^ Our results, therefore, support the use of TWO2 in the management of DFU to dramatically improve serious, painful, as well as costly patient outcomes. Equally important, this therapy is self-administered in the comfort of the patient's own home and does not require frequent visits to a specialized unit for such care.

Due to the real-world nature of the data, this study also included background use of other adjunctive therapies (NPWT, SS, and GF). For C1 (TWO2 vs. NO TWO2), the use of TWO2 was additive to other adjunctive therapies. However, in C2, when patients received TWO2 ONLY or OTHER TX ONLY (including SS, NPWT, and GF), the TWO2 ONLY group still demonstrated a meaningful reduction in the proportion of patients with hospitalization (6.9% vs. 58.8%, *p* < 0.0001) and amputation (13.8% vs. 35.3%, *p* = 0.016) at 360 days.

This suggests that TWO2 confers a significant benefit alone compared with other adjunctive therapies. These findings demonstrate the real-world, patient-centric clinical value of TWO2 both as adjunctive therapy and as a potential alternative to other advanced wound care modalities.

To our knowledge, few other studies have evaluated the impact of adjunctive modalities, including TOT, on near-term hospitalizations and amputations. In a retrospective uncontrolled chart review evaluating the impact of another type of TOT device on a variety of wound types, the overall amputation rate for patients treated with TOT was 2.4%.^21^ Although lower than observed for patients in this study (9–14% across cohorts treated with TWO2), comparisons are difficult to make due to the heterogeneity of wound types in the former study.

Furthermore, while our average DFU duration was ∼6 months, the majority of all wounds in the Copeland study were 3 months or less in duration.^21^ Nonetheless, other controlled studies and several recent RCTs have demonstrated improved healing rates and time to closure of DFUs when using TOT as compared with controls.^15,22–24^

In a sham-controlled, double-blinded RCT on the same TWO2 therapy explored in this study, Frykberg *et al.* found that 56% of TWO2 patients achieved 100% healing at 12 months (vs. 27% in the sham arm, *p* = 0.013) and only a 5% amputation rate at 1 year from enrollment.^15^ Our analysis complements Frykberg *et al.* by showing a significant reduction in hospitalizations and amputations, also at 12 months, in a large, real-world patient population with background use of adjunctive therapies. Taken together, the value of TWO2 is clearly demonstrated and warrants close consideration of its foundational role in the treatment of DFU.

Our overall 12-month amputation rate for these chronic DFU patients was 28%, consistently and significantly lower in each of the analyzed cohorts that had used TWO2. The 1-year rates of amputation seen in the cohorts without TWO2 (NO TWO2: 41% unmatched, 31% matched; OTHER TX ONLY: 35%) are fairly comparable with that seen (42.3%) in another recent retrospective study by Blumberg and Warren that also included VA hospitals.^25^

Similar to our matched NO TWO2 cohort, a 2020 meta-analysis of 21 studies and 6,505 patients by Lin *et al.* demonstrated on average that nearly 31% of patients with DFU receive amputations.^26^ Another recent study from the VA indicated that there was an increase in the rate of amputations in veterans during the years 2008 to 2018.^27^ Interestingly, these data are derived from years overlapping our own patient data and show that the increase in rates came primarily from increases in toe- and transmetatarsal-level amputations.

While our data did not categorize specific levels of amputations performed, modern limb-salvage practice has a relatively low threshold for such minor amputations in the presence of deteriorating diabetic foot wounds.^28^ This is reflected in recent nation-wide increases in the rate of diabetes-related minor amputations in the United States, manifested as a 50% increase in the total amputation rate in the years 2009–2015.^29^

The 1-year rate of amputation in our analysis may be the result of several additional factors. First, patients in this study may have had existing, nonhealing wounds before first documentation in their medical records. Second, patients with DFU in this study had high rates of PAD (77–86% across cohorts) as well as prior amputation (27–45% across cohorts). Several studies have shown that PAD and prior amputation are important risk factors for subsequent amputation in DFU.^27,30^ The patients in our study with high rates of PAD and prior amputation were therefore at higher risk for further amputation.

Renal insufficiency and end-stage renal disease are common complications in diabetes and are often considered to be predictors of failure to heal. As indicated in Table 1, 90 (99%) patients in the TWO2 group had any stage of kidney disease, with almost half having Stage III kidney disease. In contrast, 92 (83%) patients in the No TWO2 group had any stage of kidney disease, with 21 (18.9%) patients within this cohort requiring dialysis. Nonetheless, when including kidney disease stage as a covariate along with treatment in the analysis for both Hospitalization and Amputation outcomes, the kidney disease stage term was nonsignificant at *p* < 0.05 [Hospitalization *p* = 0.8218, Amputation = 0.1004].

We also recognize and acknowledge that HbA1c levels were ∼7–10% higher in our NO TWO2 cohorts (Table 1). Nonetheless, glycohemoglobin levels have long been found to be an inconsistent risk factor for DFU healing as well as risk for amputation. Accordingly, the aforementioned meta-analysis on risk factors for amputation confirmed that HbA1c level in DFU patients does not affect the incidence of amputation.^26^

Our study has several limitations inherent to any retrospective cohort analysis. First, we lacked control over the data at the time of documentation. For instance, the analysis could be impacted by missing data over the observation period. To manage missing data, we used the LOCF method and conservatively assumed that any patient without follow-up data suggesting a hospitalization or amputation during the 360-day period was categorized as not hospitalized or not amputated. Similarly, due to the nature of medical records, we did not collect data on mortality or level of amputation, as neither was reliably captured within the medical records.

Second, medical records do not capture or account for compliance with prescribed treatments. While patient compliance with TWO2 (and NPWT) is therefore unknown, this uncertainty is also reflective of and generalizable to a real-world population of patients with DFU. Third, our study does not evaluate wound healing, but rather measures outcomes in the form of hospitalizations and amputations. Fourth, the medical records follow individual patients, not specific wounds. So, it is possible that a single patient could have additional wounds that contribute to the outcomes of analysis.

Finally, treatment selection was based on the clinical judgment of the wound care physician at the time of treatment, which cannot be ascertained through a retrospective chart review, although patients at both treatment facilities had access to all treatments, including TWO2.

This study also demonstrates the benefit of TWO2 across a spectrum of DFU severities. Although most of the patients in this study were categorized as Wagner 1 and 2 upon documentation of their wounds, 20% of analyzed patients (41 of 202) were classified as Wagner 3 and 4 with more severe wounds, reflecting the real-world composition of this VA patient population.

With all severities considered, TWO2 demonstrates a statistically significant benefit over NO TWO2 in reduced incidence of hospitalization and amputation. Specifically, we found a nearly fivefold increased association with amputation and ninefold increased association with need for hospitalization in those patients who did not receive TWO2 compared with those who did.

## Conclusion

The results of this study demonstrate that home-based cyclical pressurized topical wound oxygen therapy, when used with or without other adjunctive treatments, is associated with significantly reduced frequency of wound-related hospitalization and amputation for patients afflicted with DFU. Hospitalizations and amputations are not only concerning patient outcomes affecting both morbidity and mortality, they are also costly complications that contribute to the significant overall cost burden of DFU on health care resources.^4,5^ By inference, therefore, cyclical pressurized topical wound oxygen therapy would likely be associated with important quality of life and health economic benefits.

## Data Sharing

The study protocol will be made available on request from the corresponding author. Deidentified individual participant data that underlie the results reported in this article will be made available with requests accepted immediately after publication, for proposals that set out to achieve aims specified in a methodologically and scientifically sound protocol, and where any mandated VHA approval requirements are met.

## About the Authors

**Jessica Izhakoff Yellin, AB,** is a Director at Health Advances, a strategy consulting firm focused exclusively on the health care industry. **Julia A. Gaebler, PhD** was a Partner at Health Advances. **Frank F. Zhou, BA, Olivia Novins, BA, Amelia Ockert, BA,** were all Analysts at Health Advances, and **Darcy Krzynowek, BA** is a Vice President of Health Advances. **Timothy Niecko, MS,** is President, Niecko Health Economics LLC, **Matthew G. Garoufalis, DPM** is a Podiatrist at the Jesse Brown VA Medical Center, Chicago, Illinois, **Aliza M. Lee, DPM** is a Podiatrist at the Salem Veterans Affairs Medical Center, Salem, Virginia, **Robert G. Frykberg, DPM, MPH, FFPM(Glasg), FRSM** is the Medical Director of DM Prevent Diabetic Foot and Wound Centers, and former Chief of the Podiatry section at the Phoenix Veterans Affairs Medical Center in Phoenix, Arizona. He holds Faculty ranks as Adjunct Professor, Midwestern University Program in Podiatric Medicine as well as Honorary Professor of Podiatric Medicine at NUI Galway.

## Abbreviations and Acronyms

CDO: continuous diffusion of oxygen
CVD: cardiovascular disease
DFU: diabetic foot ulcer
GF: growth factors
HBOT: hyperbaric oxygen therapy
LOCF: last observation carried forward
NPWT: negative pressure wound therapy
PAD: peripheral arterial disease
RCT: Randomized controlled trial
SD: standard deviation
SOC: standard of care
SS: skin substitutes
TOT: topical oxygen therapy
TWO2: Topical Wound Oxygen
VA: Veterans Affairs
